# Methylprednisolone attenuates lipopolysaccharide-induced Fractalkine expression in kidney of Lupus-prone MRL/lpr mice through the NF-kappaB pathway

**DOI:** 10.1186/s12882-015-0145-y

**Published:** 2015-08-27

**Authors:** Yanwu You, Yueqiu Qin, Xu Lin, Fafen Yang, Jun Li, Suren R. Sooranna, Liao Pinhu

**Affiliations:** Department of Nephrology, Affiliated Hospital of Youjiang Medical University for Nationalities, Baise, 533000 Guangxi Zhuang Autonomous Region China; Department of Gastroenterology, Affiliated Hospital of Youjiang Medical University for Nationalities, Baise, 533000 Guangxi Zhuang Autonomous Region China; Department of Intensive Care Medicine, Affiliated Hospital of Youjiang Medical University for Nationalities, Baise, 533000 Guangxi Zhuang Autonomous Region China; Department of Surgery and Cancer, Imperial College London, Chelsea and Westminster Hospital, London, SW10 9NH UK

**Keywords:** Fractalkine, NF-κB, LPS, Methylprednisolone, MRL/lpr Mice

## Abstract

**Background:**

Fractalkine (FKN) is involved in the occurrence and development of human lupus nephritis. It is known to be upregulated by lipopolysaccharide (LPS) as a stimulus *in vivo*. MRL/lpr mice have been used as an *in vivo* model to study lupus nephritis. Methylprednisolone (MP) is used widely in the clinical treatment of progressive glomerular diseases such as lupus nephritis. The aim of this study is to explore the mechanism of LPS induced FKN expression and to determine whether other molecular mechanisms contribute to the signaling pathway of MP action in MRL/lpr mice.

**Methods:**

Forty-eight female MRL/lpr mice at 12 weeks of age were randomly distributed into six groups. Each group received various treatments for 8 weeks by receiving twice weekly intraperitoneal injections of (1) MP (MP-treated mice), of (2) SC-514 (SC-514-induced mice), of (3) normal saline and a single injection of LPS (LPS-induced mice), of (4) MP and a single injection of LPS (LPS + MP mice), of (5) SC-514 and a single injection of LPS (LPS + SC mice) and of (6) normal saline (control mice). One-way ANOVA was used for data analysis and *P* value <0.05 was considered statistically significantly.

**Results:**

The expression of FKN and NF-kappaB p65 mRNA was detected by qPCR. The expression of FKN protein and the activation of NF-kappaB p65 were detected by immunohistochemistry and western blots respectively. The expression of FKN in the kidney of LPS induced mice was significantly increased and this was mediated by increased expression of NF-κB p65 and an increase in NF-kappaB phospho-p65. MP reduced proteinuria and ameliorated the renal damage in MRL/lpr mice. MP as well as the NF-kappaB inhibitor, SC-514, inhibited the LPS-induced increase of expression of FKN and the activation of NF-kappaB.

**Conclusions:**

The results indicate that MP attenuates LPS-induced FKN expression in kidney of MRL/lpr mice through the NF-kappaB pathway.

## Background

Systemic lupus erythematosus (SLE) is a systemic autoimmune disease. Renal involvement, namely lupus nephritis, is a common and potentially lethal complication of SLE. It involves the production of pathogenic autoantibodies, hypocomplementemia, deposition of immune complexes, inflammation and renal damage [1]. The morphologic changes in lupus nephritis include a spectrum of vascular, glomerular and tubulointerstitial lesions. Glomerular injury comprises mesangial hypercellularity and matrix accumulation as the mesangial pattern, with leukocyte accumulation, endothelial cell injury and endocapillary proliferation as the endothelial pattern, and a nonexudative, nonproliferative capillary wall lesion as the epithelial pattern [2]. Although the pathogenesis of lupus nephritis has yet to be determined, increasing lines of evidence have indicated a potential role of cytokines in the development and progression of lupus nephritis [3].

MRL/MpJ-lpr/lpr (MRL/lpr) mice develop spontaneous autoimmune diseases and the main histopathological features of the glomerular lesions resemble those shown in human lupus nephritis. These serious glomerular diseases that develop spontaneously in MRL/lpr mice are commonly associated with cytokine abnormalities. FKN is a unique member of the CX3C superfamily of chemokines [4] and acts as a chemotatic agent. It also functions as an adhesion molecule and mediates immune injury and is involved in the occurrence and development of renal diseases [5–7], including lupus nephritis [8]. FKN can be synthesized by a variety of progressive glomerular diseases [9–11] in lupus-prone MRL/lpr mice [8, 11, 12]. It is produced as an intracellular precursor protein and transported to the cell surface as a mature protein after being N-glycosylated [13]. FKN is significantly expressed in the glomeruli of 12-week-old MRL/lpr mice [14]. N-terminal-truncated analogs acting as FKN antagonists can reduce glomerular damage and interstitial mononuclear cell infiltration [14]. FKN is produced secondary to the deposition of immune complexes [14]. These results indicate that FKN may be able to modify the progression of renal damage in MRL/lpr mice.

NF-κB also has been shown to be involved in lupus nephritis [15]. LPS can induce moderate albuminuria and aggravate glomerular nephritis in MRL/lpr mice [16]. LPS induces expression of inflammatory cytokines and activation of the NF-κB pathway [17], FKN mRNA and protein expression and these have been shown to increase in mesangial cells stimulated with LPS [18]. FKN has also been shown to induce proliferation of mesangial cells [19]. NF-κB may thus be an important target for an anti-inflammatory approach to treat LPS-induced alterations of FKN expression.

Current induction therapy for some severe forms of lupus nephritis such as Class III/IV are various combinations of glucocorticoids with other agents [20] because of their important anti-inflammatory and immunosuppressive effects [21, 22]. In human lupus nephritis, the expression of FKN in glomeruli correlates with the histopathologic activity index whereby the glomerular FKN expression has a tendency to decrease with glucocorticoid therapy [8]. MP is one of the glucocorticoids used as an initial starting point of treatment for lupus nephritis. In respiratory epithelial cells, the NF-κB pathway regulates the expression of FKN, and glucocorticoids have been shown to inhibit FKN expression in a glucocorticoid receptor-dependent (RU486 sensitive) manner [23]. The mechanisms by which MP inhibits inflammation and the signaling pathways in lupus nephritis, such as the FKN and NF-κB pathways, are not fully understood.

This study uses a molecular biology-based approach to demonstrate the expression of FKN and activation of NF-κB in MRL/lpr mice treated with LPS. In addition, the molecular mechanisms and the pathways involved in LPS-induced FKN expression in lupus nephritis and the molecular mechanisms by which MP modulates lupus nephritis are explored.

## Methods

### Animals

MRL/lpr mice weighing 18-21 g were purchased from the Model Animal Research Center of Nanjing University (Nanjing, China). Mice were fed under conditions free of specific pathogens at 22-25 °C and kept in an environment of 40-60 % relative humidity in the Animal Research Institute of Youjiang Medical University for Nationalities. All procedures involving mice were approved by the Committee on the Ethics of Animal Experiments of Youjiang Medical University for Nationalities and were carried out in accordance with the National Institute of Health guidelines.

### Experimental protocol

Forty-eight female MRL/lpr mice at 12 weeks of age were randomly distributed into six groups that received intraperitoneal injections as follows:

1. twice weekly injections for 8 weeks of MP (25 mg/kg body weight, Pfizer, Belgium) - MP-treated mice
2. twice weekly injections for 8 weeks of SC-514 (25 mg/kg body weight, SML0557 from Sigma-Aldrich, USA). SC-514 is a cell-permeable, potent and selective ATP competitive inhibitor of NF-κB kinase-2 that specifically blocks NF-κB-dependent gene expression. - SC514-treated mice
3. twice weekly injections for 8 weeks of normal saline and a single injection of LPS (5.0 mg/kg body weight, L2880, Sigma-Aldrich, USA) 8 h prior to sacrifice as described previously [24] - LPS-induced mice
4. twice weekly injections for 8 weeks of MP and a single injection of LPS 8 h prior to sacrifice - LPS + MP mice
5. twice weekly injections for 8 weeks of SC-514 and a single injection of LPS 8 h prior to sacrifice - LPS + SC mice
6. twice weekly injections for 8 weeks of normal saline - control mice.

### Assessment of urine protein and renal function

Urine samples were collected using metabolic cages for 24 h before sacrifice. Urine protein was measured as described previously [25]. Blood serum levels of urea nitrogen (BUN) and creatinine (Cr) were determined at 20 weeks when all mice were subsequently sacrificed 8 h after the last injection of LPS or saline as described previously [25]. Renal cortical samples were harvested at this time.

### Histopathological analysis

Tissue samples were fixed with 10 % formalin in 0.01 mol/L phosphate buffer (pH 7.2) and then embedded in paraffin for histopathology. The slides were stained with periodic acid-silver methenamine (PASM) for examination under a light microscope. The examination of renal pathology was performed in a blinded fashion by a pathologist.

### RNA isolation and real-time PCR assays

Total RNA of renal cortices was extracted using TRIzol reagent (Invitrogen, USA) according to the manufacturer’s instructions. First-strand cDNA was then made from the total RNA using M-MLV reverse transcriptase (C28025, Invitrogen, Shanghai, China) with random primers. The expression levels of FKN and p65 were measured by real-time qPCR using SYBR Green I (204143, Qiagen, Germany) through forced denaturation at 95 °C for 30 s and 40 cycles of denaturation at 95 °C for 10 s, annealing and extension at 60 °C for 30 s each. Primer sequences were designed using the Primer Premier 3.0 program. The optimal reference genes were EEF1A1 and GAPDH in MRL/lpr mice, determined by geNorm 3.5 software as described previously [26]. Primer sequences (Invitrogen, USA) were as follows: mice FKN forward 5′- CGACAAGATGACCTCACGAA -3′, reverse 5′- CTGTGTCGTCTCCAGGACAA -3′(NM_009142.3); mice P65 forward 5′- TAAGCCGTACACAGCCACTG -3′, reverse 5′ - CCAGGTAAATGGCTGCAGAT -3′ (NM_010908.4); mice EEF1A1 forward 5′- TAGACGAGGCAATGTTGCTG -3′, reverse 5′ - AGCGTAGCCAGCACTGATTT -3′(NM_010106.2); mice GAPDH forward 5′- AACTTTGGCATTGTGGAAGG -3′, reverse 5′ - GGATGCAGGGATGATGTTCT -3′(NM_008084.2). The comparative gene expression was calculated by 2^-△△Ct^ method as described previously [27].

### Immunohistochemistry

For immunohistochemistry (IHC), formalin-fixed and paraffin embedded renal sections were prepared as described previously, and then incubated in 3 % hydrogen peroxide for 15 min and normal goat serum for 10 min to block endogenous peroxidase. The sections were incubated with either anti-FKN antibody (ab25088, Abcam Ltd, Hong Kong, 1:200 dilution) or anti-NFκB p65 antibody (ab31481, Abcam Ltd, Hong Kong, 1:100 dilution) overnight at 4 °C and then incubated with goat polyclonal secondary antibody to rabbit IgG-H&L (HRP) (ab6721, Abcam Ltd, Hong Kong, 1:500 dilution) for 30 min at 37 °C. Normal rabbit serum served as a negative control. To compare the expression levels of FKN and p65 in glomerular cells by IHC, staining intensity was evaluated semiquantitatively according to the previous paper [10]. Forty or more glomeruli were examined on each slide and assigned values of staining intensity from 0 to 3+. An intensity score was calculated as: (% glomeruli intensity (negative) × 0) + (% glomeruli intensity (trace intensity) × 0.5) + (% glomeruli (1+) intensity × 1) + (% glomeruli (2+) intensity × 2) + (% glomeruli (3+) intensity × 3). The values typically ranged from 0 to a maximum of 300.

### Western blot analysis

Aliquots of total kidney homogenate from individual animals were diluted in lysis buffer (50 mM Tris–HCl, pH 7.4, 150 mM NaCl, 1 % Triton X-100, 0.1 % SDS, 2 mM EDTA, 0.1 mM EGTA, 5 mM NaF, 1 mM Na_3_VO_4_, 5 mM Na_2_PO_4_ and 1 × proteinase inhibitor cocktail (Beyotime Institute of Biotechnology, China) to a final protein concentration of 2 μg/μL. Western blot analysis was conducted and quantified as described by Müller et al. [28]. The following antibodies were used: rabbit anti-FKN antibody (ab25088, Abcam Ltd, Hong Kong, 1:100 dilution) or anti-NFκB p65 antibody (ab31481, Abcam Ltd, Hong Kong, 1:200 dilution) or anti-NF-κB phospho p65 antibody (ab28810, Abcam Ltd, Hong Kong, 1:100 dilution) to evaluate the activity of NF-κB pathway, and mouse anti-beta actin monoclonal antibody (AA128, Beyotime Institute of Biotechnology, China, 1:500 dilution) followed by the goat polyclonal secondary antibody to rabbit IgG-H&L (HRP) (ab6721, Abcam Ltd, Hong Kong, 1:1000 dilution) or anti-mouse IgG-H + L (A0216, Beyotime Institute of Biotechnology, China, 1:1000 dilution). The image of western blots was scanned by Quantity One software and the original intensity of each specific band was quantified with freeware image analysis software, NIH Image (National Institute of Health, Bethesda, Md., USA).

### Statistical analysis

Data are reported as means ± SE for normally distributed data and median (range) for nonparametric data. The comparisons of gene expression levels between groups were performed by using the one-way ANOVA for parametric data, F-test for equality of variances and Newman-Keuls test for heterogeneity of variance. All analyses were conducted with SPSS software, version18.0. *P* value < 0.05 was considered statistically significantly.

## Results

### MP reduces proteinuria and renal function defects in MRL/lpr mice

**Fig. 1:**
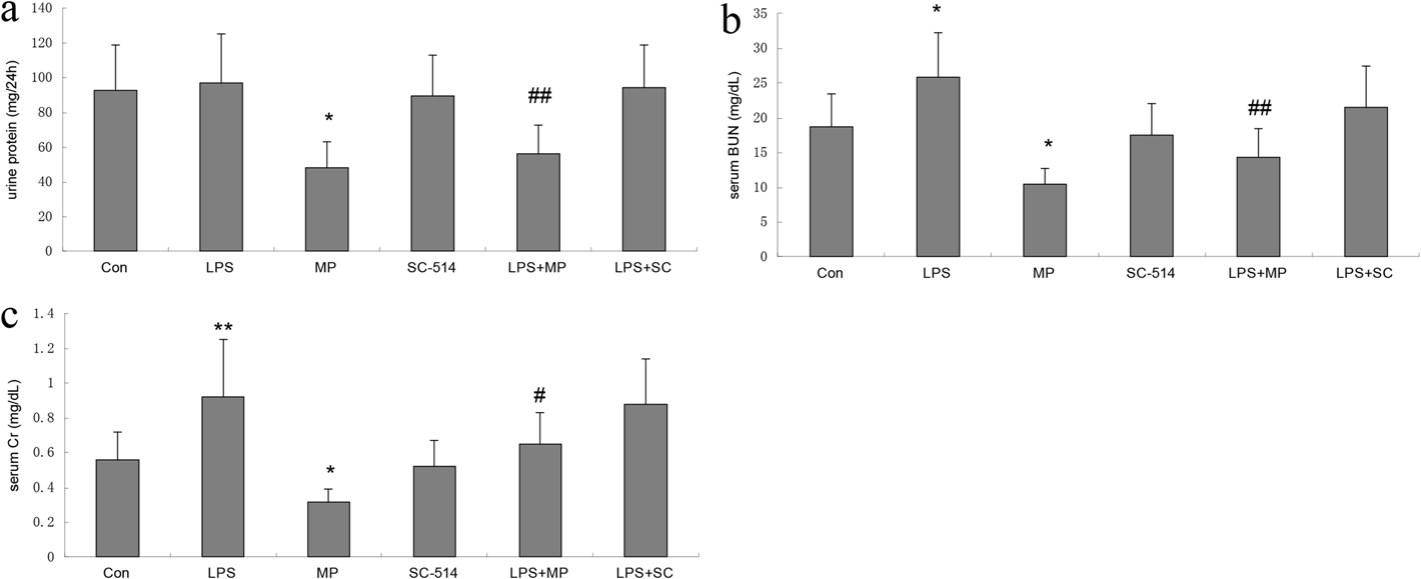
Proteinuria and renal function in MRL/lpr mice. **a** Urine protein levels in MRL/lpr mice. *P < 0.05 when compared with control mice; ## *P* < 0.01 when compared with LPS mice. **b** Serum levels of BUN in MRL/lpr mice. *P < 0.05 when compared with control mice; *P < 0.05 when compared with control mice; ## *P* < 0.01 when compared with LPS mice. **c** Serum levels of Cr in MRL/lpr mice. **P < 0.01 when compared with control mice; *P < 0.05 when compared with control mice; # *P* < 0.05 when compared with LPS mice

MRL/lpr mice showed moderate proteinuria and renal function defects at 20 weeks. Proteinuria in 20-week-old MRL/lpr mice was 92.5 ± 26.3 mg/24 h. An intraperitoneal injection of LPS did not induce proteinuria (96.8 ± 32.6 mg/24 h) but MP was able to reduce this level significantly (48.3 ± 22.8 mg/24 h; *P* < 0.05). Compared with LPS-induced MRL/lpr mice, proteinuria in LPS + MP-treated MRL/lpr mice was decreased (56.2 ± 23.6 mg/24 h; *P* < 0.01; Fig. 1a). There was no significant difference of proteinuria between SC-514-treated MRL/lpr mice and control mice, and between LPS + SC-treated MRL/lpr mice and LPS-induced mice. The LPS-induced MRL/lpr mice showed significantly increased serum levels of BUN and Cr, and there was a dramatic improvement in renal function in the MP-treated MRL/lpr mice. There was no significant difference of serum BUN and Cr between SC-514-treated MRL/lpr mice and control mice, and between LPS + SC-treated MRL/lpr mice and LPS-induced mice (Fig. 1b and c).

### MP ameliorates the severe renal lesions in MRL/lpr mice

**Fig. 2:**
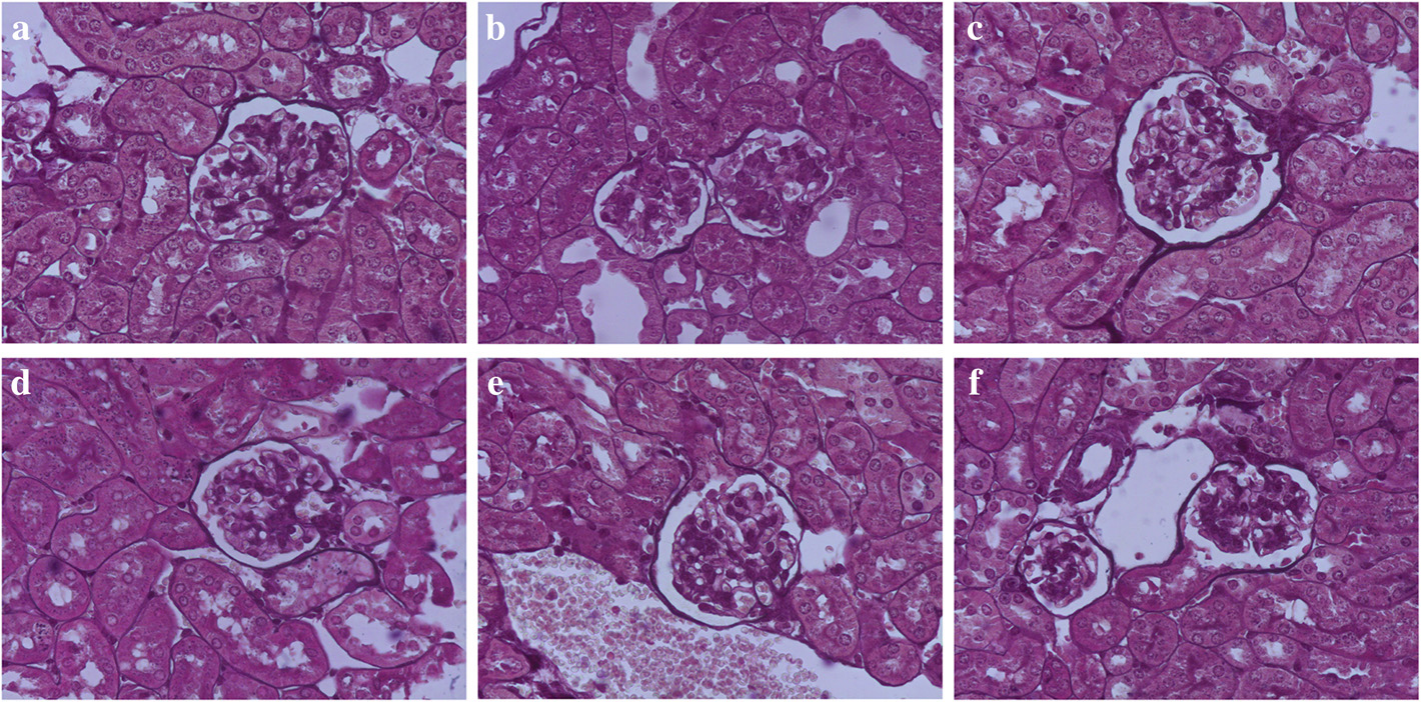
Renal histopathological staining of MRL/lpr mice by PASM. **a** Control mice show widening of the mesangial region, increased mesangial matrix, a mesangial cells proliferation degree of between 25-50 % and formation of cellular crescent. **b** LPS-induced mice show mesangial cells proliferation degree of more than 50 %, and inflammation. **c** MP-treated mice show mild mesangial matrix and mesangial cells proliferation. **d** SC-514-treated mice show increased mesangial matrix, a mesangial cells proliferation degree of between 25-50 %. **e** LPS + MP-treated mice show mild mesangial matrix and mesangial cells proliferation. **f** LPS + SC-treated mice show mesangial cells proliferation degree of more than 50 % (original magnification, ×400)

At the age of 20 weeks, the renal cortex of MRL/lpr mice showed widening of the mesangial region, increased mesangial matrix, proliferation of mesangial cells (the degree of proliferation was between 25 and 50 %), lobulation of the glomerulus and formation of partial cellular crescents. These changes are indicative of renal structural damaged. The renal cortex of SC-514-treated MRL/lpr mice also showed a similar extent of mesangial cell proliferation and fusion of podocytes, but no formation of partial cellular crescents and less electron-dense deposits in the mesangial region. The renal cortex of LPS-induced and LPS + SC-treated MRL/lpr mice showed mesangial cell proliferation (proliferation degree of more than 50 %), with endothelial cell proliferation, vascular loop compression, partial occlusion, interstitial inflammation and infiltration of lymphocytes, monocytes and eosinophils. However, the renal cortex of MP and LPS + MP-treated mice showed a reduction in mesangial matrix, lowered mesangial cell proliferation (with a proliferation degree of between 25 and 50 %) and no crescent formation and interstitial inflammation (Fig. 2).

### MP and SC-514 inhibit gene expression of FKN and NF-κB in MRL/lpr mice

**Fig. 3:**
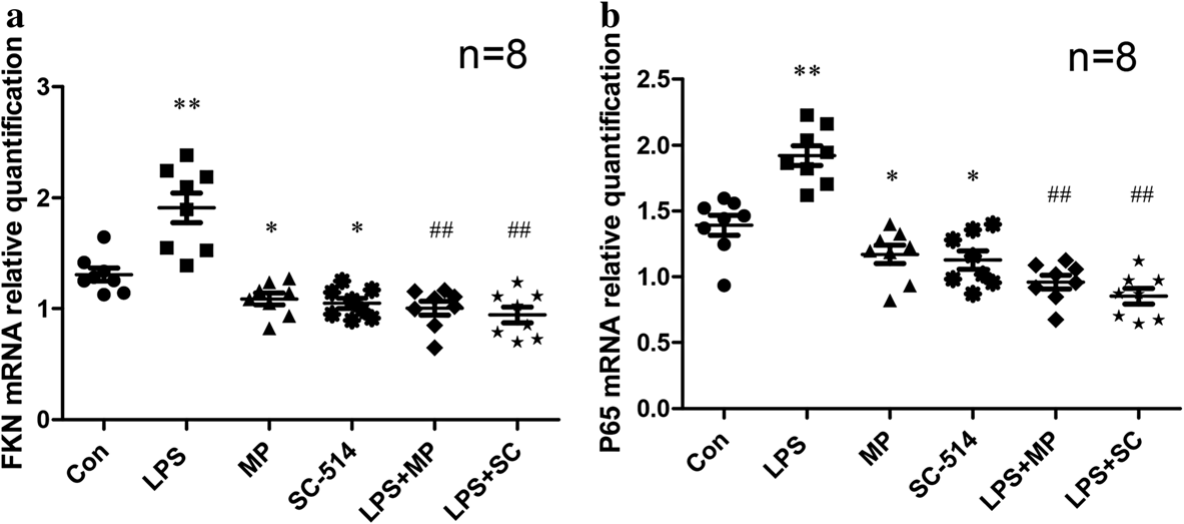
Expression of FKN and NF-κB p65 mRNA in renal cortex. The semiquantification analysis of (**a**) FKN and (**b**) p65 mRNA by qPCR. **P < 0.01 when compared with the control mice; *P < 0.05 when compared with the control mice; ## *P* < 0.01 when compared with LPS-induced mice

The semiquantification analysis of FKN and NF-κB p65 mRNA in renal cortex was compared between the groups of mice by real-time PCR. The relative FKN and p65 mRNA levels (normalized to EEF1A1 and GAPDH mRNA) in renal cortex of LPS-induced mice were significantly greater than those in control mice. The FKN and p65 mRNA levels of MP and SC-514 mice were down-regulated compared with control mice. The FKN and p65 mRNA levels of LPS + MP-treated and LPS + SC-treated mice were down-regulated compared with LPS-induced mice (Fig. 3).

### Localization and the expression of renal FKN and p65 Protein in MRL/lpr mice

**Fig. 4:**
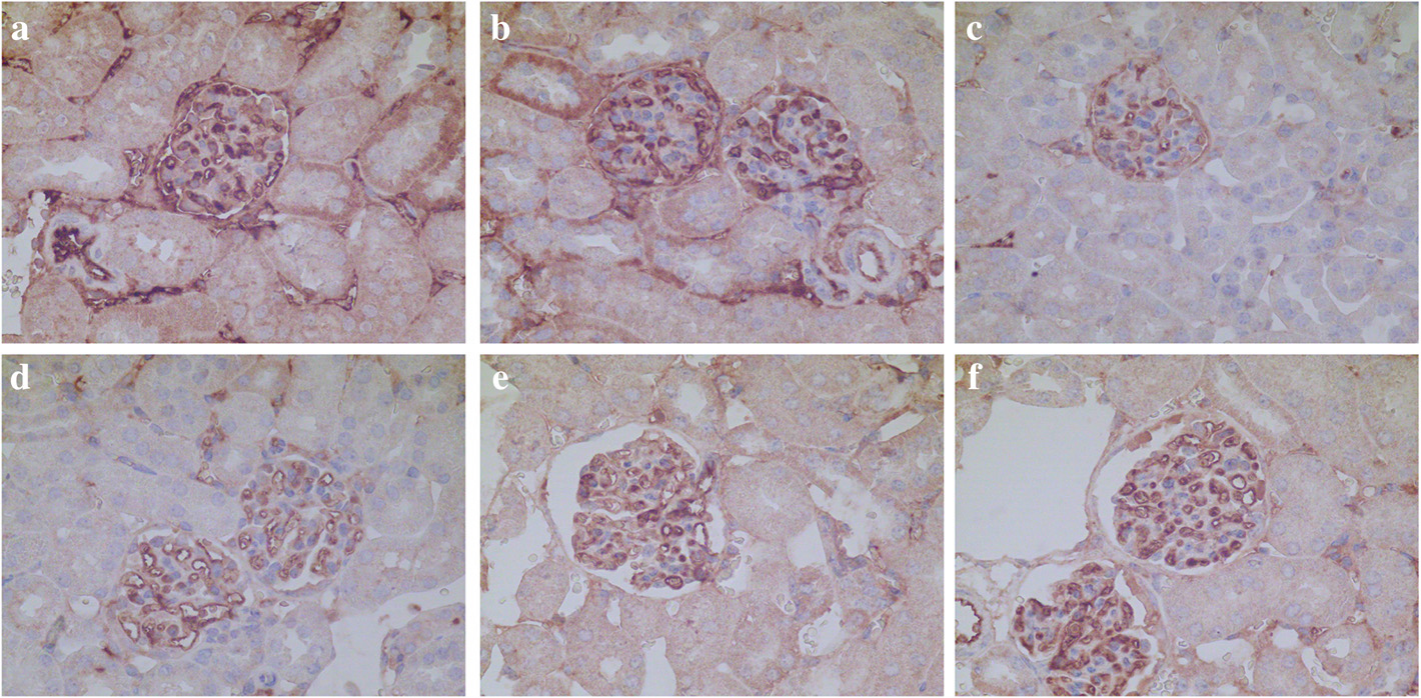
Immunohistochemistry for FKN within glomerular lesions in MRL/lpr mice. **a** Control mice, **b** LPS-induced mice, **c** MP-treated mice, **d** SC-514-treated mice, **e** LPS + MP-treated mice, **f** LPS + SC-treated mice (original magnification, ×400)

**Fig. 5:**
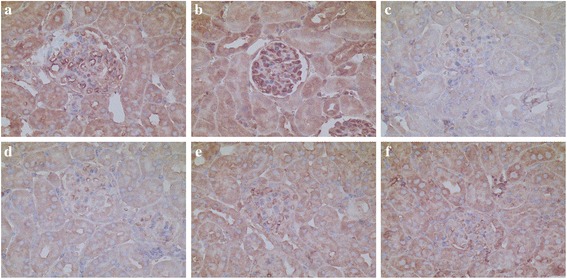
Immunohistochemistry for p65 within glomerular lesions in MRL/lpr mice. **a** Control mice, **b** LPS-induced mice, **c** MP-treated mice, **d** SC-514-treated mice, **e** LPS + MP-treated mice, **f** LPS + SC-treated mice (original magnification, ×400)

In kidney sections of MRL/lpr mice stained by IHC, the expression of FKN and p65 protein was seen mainly in the cytoplasm of podocytes, endothelial cells in glomerular and some renal tubular epithelial cells (Figs. 4 and 5). The expression of FKN and p65 protein was compared between the different groups of mice. According to semiquantitative evaluation, the intensity scores of FKN were 142.5 ± 38.7, 196.7 ± 36.5, 96.3 ± 21.6, 99.3 ± 21.5, 102.8 ± 32.4 and 118.7 ± 29.5 in control, LPS-induced, MP-treated SC-514-treated, LPS + MP and LPS + SC mice respectively. The intensity scores of p65 were 98.2 ± 32.5, 143.8 ± 44.2, 62.3 ± 18.5, 58.5 ± 17.7, 75.2 ± 21.3 and 74.5 ± 22.6 in control, LPS-induced, MP-treated SC-514-treated, LPS + MP and LPS + SC mice respectively. There was increased expression of glomerular FKN and p65 protein in LPS-induced mice (*P* < 0.01 and *P* < 0.05 respectively) when compared to control mice, a decreased expression in MP-treated mice compared to control mice (*P* < 0.05) and a decreased expression in LPS + MP and LPS + SC mice compared to LPS-induced mice (*P* < 0.01 and *P* < 0.05 respectively).

### MP as well as SC-514 inhibit FKN renal protein expression and NF-κB activation in MRL/lpr mice

**Fig. 6:**
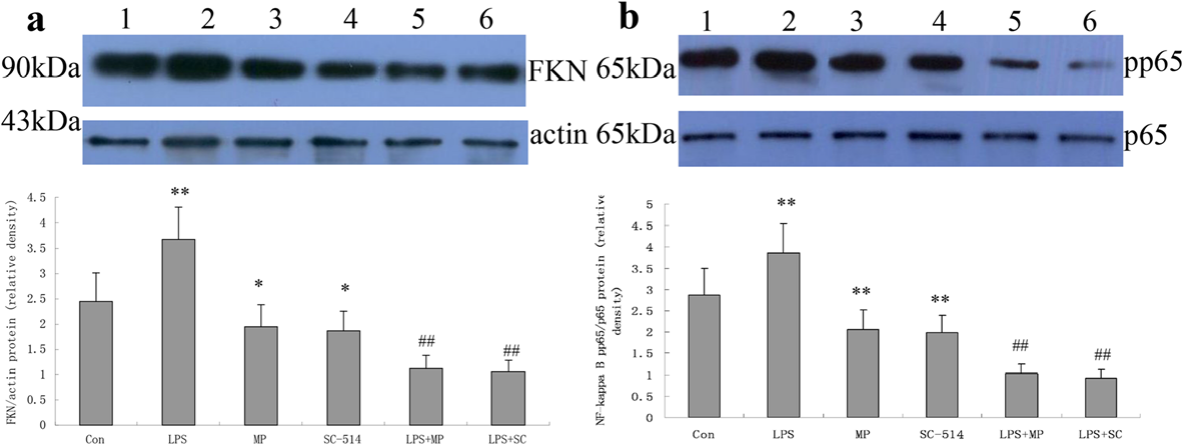
Expression of FKN and NF-κB p65 protein in renal cortex. The relative density of **a** FKN/beta-actin and **b** phospho p65 (pp65)/p65 protein. Lanes: (1) control mice, (2) LPS-induced mice, (3) MP-treated mice, (4) SC-514-treated mice, (5) LPS + MP-treated mice and (6) LPS + SC-treated mice. ** *P* < 0.01 when compared with control mice; * *P* < 0.05 when compared with control mice; ## *P* < 0.01 when compared with LPS-induced mice

Western blot analysis showed the expression of FKN and activation of NF-κB by testing for phospho p65 in renal cortex of MRL/lpr mice. As shown in Fig. 6, LPS stimulated the expression of FKN and phospho p65 protein with MP or SC-514 suppressing LPS-induced expression of FKN and phospho p65 protein in MRL/lpr mice kidney. These results are consistent with the data relating to FKN and p65 mRNA expression and the immunohistological findings.

## Discussion

The results demonstrate that LPS induced the expression of FKN and activation of NF-κB in renal cortex of MRL/lpr mice and that MP significantly reduced proteinuria, ameliorated renal function, severity of renal pathology and LPS-induced kidney injury in MRL/lpr mice. MP downregulated the LPS-induced FKN expression and NF-κB activation in kidney of MRL/lpr mice, consistent with the role of the NF-κB inhibitor, SC-514.

Chemokines are known to be involved in the lupus disease process. CCL2 (also known as monocyte chemotactic protein-1, MCP-1) [29] and CXCL10 are good biomarkers to indicate the activity of lupus nephritis [30] and the potential flares in SLE [31]. CCL2 and CXCL10 are known to be raised in the blood of SLE patients [32]. Compared to treated patients, expression of CCL2 and CXCL10 mRNA in untreated SLE patients’ blood were significantly higher. This indicates that chemokines are potential therapeutic targets in SLE patients [33]. Kulkarni et al. [34] used the mNOX-E36 to inhibit CCL2 in MRL/lpr mice and found prolonged survival associated with the improvement of lupus nephritis. In addition, CCL2 antagonists have been shown to be beneficial in the treatment of lupus nephritis. Kassianos et al. demonstrated recently the role of FKN and its receptor CX3CR1 in the recruitment and retention of human kidney dendritic cells (DCs), and showed the TGF-β-producing, CX3CL1-expressing CD1c^+^ DCs were recruited and retained within the tubuleinterstitium during fibrotic kidney disease via proximal tubular epithelial cell (PTEC)-derived FKN [35].

Expression of FKN mRNA and protein significantly increased in the renal cortex of the LPS-induced group in lupus mice, which shows that LPS can induce FKN expression in MRL/lpr mice. These data are consistent with the results seen in mesangial cells *in vitro* [18]. MP inhibited significantly the expression of FKN mRNA and protein in renal cortex of MRL/lpr mice. These effects correlated with a reduction in proteinuria as well as amelioration of renal function and renal pathology. SC-514 is a selective and reversible inhibitor of IKKβ (IKK-2), affecting NF-κB nuclear import/export as well as the phosphorylation and transactivation of p65. SC-514 was used to suppress the NF-κB activity in this study. SC-514 also significantly inhibited expression of FKN mRNA and protein in renal cortex of MRL/lpr mice. The results suggest that MP as well as SC-514 can inhibit the increased expression of FKN induced by LPS in MRL/lpr mice. However, the effect of SC-514 was not paralleled to that of MP on proteinuria, renal function and glomerular proliferation in MRL/lpr mice. Therefore, in addition to NF-κB pathway, there may be some other mechanisms involved in the treatment of lupus nephritis that needs to be explored.

IκBs, which regulate the nuclear translocation of NF-κB, are critically associated to the differentiation of B cells and with the auto-antibodies produced during progression of SLE disease [36]. Activation of NF-κB in renal cortex in MRL/lpr mice was detected in this study. The significant increase in expression of NF-κB p65 and activation of NF-κB induced by LPS probably contribute to the progression of glomerular lesions in the lupus nephritis model. MP treatment significantly inhibited expression of NF-κB p65 and activation of the NF-κB pathway, which was confirmed by the use of the NF-κB inhibitor, SC-514. These effects are likely to be associated with expression of FKN mRNA and protein. The other chemokine member, CXCL12 and its receptor CXCR4, have been shown to be markedly elevated in infected lupus mice via activation of the NF-κB signaling pathway [37]. The data presented here are consistent with previous observations summarizing the cytokine-suppressing effects of NF-κB inhibitors resulting in a reduced FKN expression during inflammation-associated diseases [38]. Accordingly, these results are consistent for a central mechanism of MP in modulation of FKN expression by suppressing the activation of NF-κB during lupus nephritis.

## Conclusions

This study confirms early findings that LPS-induced expression of FKN in the kidney of MRL/lpr mice is mediated through the NF-κB pathway with the attenuation of LPS-induced FKN expression by MP being accompanied by the suppression of NF-κB activation. This leads us to conclude that the mechanism of action of MP may be partly specific to the FKN gene and that it mediates its suppressive effects through the NF-κB-dependent pathway in lupus nephritis. With respect to whether FKN contributes to the disease progression, more studies are required on the FKN-CX3CR1 system in order to explore therapeutic potential in lupus nephritis.
